# Association Between Smoking and Pain, Functional Disability, Anxiety and Depression in Patients With Chronic Low Back Pain

**DOI:** 10.3389/ijph.2023.1605583

**Published:** 2023-03-07

**Authors:** Qi-Hao Yang, Yong-Hui Zhang, Shu-Hao Du, Yu-Chen Wang, Xue-Qiang Wang

**Affiliations:** ^1^ Department of Sport Rehabilitation, Shanghai University of Sport, Shanghai, China; ^2^ Department of Rehabilitation Medicine, Shanghai Shangti Orthopaedic Hospital, Shanghai, China

**Keywords:** chronic low back pain, tobacco use, psychological symptom, correlation, pain management

## Abstract

**Objectives:** Chronic low back pain (CLBP) accounts for a majority of the disability associated with LBP, which can produce long-term negative effects. This cross-sectional study aimed to investigate the association between smoking and pain, dysfunction and psychological status in patients with CLBP.

**Methods:** The 54 patients with CLBP were recruited and divided into smoking and non-smoking groups. Their pain, dysfunction, anxiety, depression, fear and quality of life were evaluated. The amount of cigarettes smoked daily was recorded.

**Results:** Significant differences in VAS, ODI, RMDQ and FABQ and the impact of LBP on life and work were found between smoking and non-smoking patients. In addition, a correlation was found between the daily cigarette smoking amount and VAS_max_, FABQ_total_, SDS and FABQ-W. Moreover, a correlation was observed between the amount of cigarettes smoked daily and the degree of impact of low back pain on work.

**Conclusion:** The study found that smoking affected the aggravation of symptoms in patients with CLBP, which indicated that patients with CLBP and people at risk of LBP should be aware of the harm caused by smoking.

## Introduction

Low back pain (LBP) is a common musculoskeletal disorder experienced by adults of all ages (1). In the US, more than 55% of adults report back pain in the past year (2). LBP is the primary cause of restricted activity, sickness absence, loss of work productivity and reduced quality of life worldwide, resulting in high healthcare costs for individuals, families, and society (3). Chronic low back pain (CLBP) accounts for a majority of the dysfunction and expenses associated with LBP. The annual total direct cost per patient with CLBP in the US is $8,386 (4). Studies have shown that exercise can relieve LBP (5–7). The risk factors for LBP are multifactorial, including hereditary factors, physical risk factors (such as gender, age and history of a back injury), psychological factors (such as long-term mental stress, anxiety and fear of activities that indicate bodily harm or pain) and unhealthy lifestyle (such as alcohol drinking and smoking) (8, 9).

Recently, the interrelationships between pain, cigarette and smoking have received considerable attention because of their prevalence, public health consequences and serious comorbidities (10). More than two-thirds of Americans with chronic pain support lifelong nicotine use (11). People with chronic pain likely smoke more than the general population (12). Furthermore, recent estimates suggest that nearly 60% of people who are addicted to tobacco meet the criteria for chronic pain (13). Previous research has shown that smokers and those who have quit smoking tend to experience more widespread and severe pain than non-smokers (14).

Smoking has been linked to headaches, trunk pain and pain in the extremities (15, 16). Despite the increased likelihood of generalized pain, studies have found a strong relationship between spinal pain and smoking (16). Current and previous heavy smoking are associated with the amount and intensity of pain sites, that is, heavy smokers have a higher chance of having more pain sites and greater pain intensity than non-smokers (14). However, research on the association between smoking and pain intensity, function, depression and fear in patients with CLBP remains limited, particularly in studies that have conducted detailed comparisons of disease activity and functional status in smoking and non-smoking patients with CLBP. This study aims to investigate the interrelationship between smoking and CLBP from multiple dimensions such as pain intensity, psychology and quality of life. In addition, this study will investigate whether the amount of cigarettes smoked daily affects the pain intensity, dysfunction, anxiety and depression degree of patients with CLBP.

## Methods

### Study Design

This cross-sectional study assessed pain, dysfunction, anxiety, depression, fear and quality of life in 54 patients with CLBP and compared the differences between smokers and non-smokers. We further examined the association between the amount of cigarettes smoked and pain, dysfunction and depression in patients with CLBP. Baseline data, including sex, age, height, weight, work status, physical activity, location of pain and duration of pain, were recorded. All patient assessment questionnaires were completed under the supervision of one researcher, and baseline data were recorded by another researcher.

### Participants

G∗Power software was used to calculate sample size (one tail; *α* = 0.05; Power = 0.95; N2/N1 = 1), which is based on a previous study of differences in pain intensity measured by VAS between smokers and non-smokers (17). The results showed that N1 and N2 were 26, and the actual power was 0.950. A total of 54 community residents in the main communities of Qingyuan Street, Yangpu District, Shanghai, were examined with an equal number of smokers and non-smokers. The inclusion criteria were: 1) age 18–65; 2) pain confined to the waist, buttocks and thighs, with or without leg pain; 3) pain intensity at worst 3 or higher on a visual analog scale; 4) CLBP for at least 3 months; 5) patients signed informed consent after receiving the purpose and method of this study. The exclusion criteria were: 1) with mental and cognitive diseases; 2) specific lumbago; 3) with neurological disorders, such as stroke and epilepsy. Standardized questionnaires were used to collect demographic information (e.g., age and gender), education, residential status, marital status, economic income, physical activity level and medical history. The comprehensive demographic variables are provided in Table 1.

**TABLE 1:** Demographic characteristics of participants (Shanghai, China. 2018–2019).

Characteristic	Smoking group (*n* = 27)	Non-smoking group (*n* = 27)	*p* Value
Age, y	29.06 (22.11, 46.09)	30.09 (25.05, 37)	0.99
Sex-male, n (%)	18 (66.7)	16 (59.3)	0.78
Height, m	171 (166, 177.8)	167.2 (161.5, 171.7)	0.14
Weight, kg	71.8 (66.9,81.1)	68.3 (55.9,74.8)	0.13
BMI, kg/m^2^	24.1 (22.6,27.4)	22.8 (20.4, 25.9)	0.18
Education levels			0.09
Junior middle school, n (%)	3 (11.1)	0 (0)	
High school, n (%)	2 (7.4)	1 (3.7)	
University, n (%)	18 (66.7)	12 (44.4)	
Postgraduate, n (%)	4 (14.8)	14 (51.9)	
Employment status			0.30
Employed part-time, n (%)	2 (7.4)	1 (3.7)	
Employed full-time, n (%)	13 (48.1)	16 (59.3)	
Student, n (%)	9 (33.3)	10 (37)	
Retired, n (%)	3 (11.1)	0 (0)	
Economy-income level			0.08
None	7 (25.9)	4 (14.8)	
Less than 3,000 ¥ per month	2 (7.4)	6 (22.2)	
3,000–5,000 ¥ per month	7 (25.9)	1 (3.7)	
5,000–10000 ¥ per month	5 (18.5)	7 (25.9)	
More than 10,000 ¥ per month	6 (22.2)	9 (33.3)	
Marital status			0.85
Unmarried, n (%)	14 (51.9)	12 (44.4)	
Married, n (%)	11 (40.7)	13 (48.1)	
Divorced, n (%)	2 (7.4)	2 (7.4)	
Occupation time, h	8 (8, 10)	8 (8, 9)	0.74
Leisure time, h	7 (6, 8)	8 (7, 8)	0.17
Moderate-intensity physical activity per week			0.35
<75 min per week, n (%)	6 (22.2)	10 (37)	
75–150 min per week, n (%)	6 (22.2)	7 (25.9)	
>150 min per week, n (%)	15 (55.6)	10 (37)	
Site of LBP			0.37
Left, n (%)	7 (25.9)	3 (11.1)	
Right, n (%)	4 (14.8)	8 (29.6)	
Middle, n (%)	9 (33.3)	8 (29.6)	
Both two sides, n (%)	7 (25.9)	8 (29.6)	
Low back pain Occurrence			0.77
Continuous, n (%)	8 (29.6)	9 (33.3)	
Interinittent, n (%)	19 (70.4)	18 (66.7)	
History of low back pain, y	4.42 (3.33, 7.58)	4.17 (3.58, 9.83)	0.92
Duration of first onset, d	7 (2, 60)	7 (2, 21)	0.43
Duration of low back pain per day, h	6 (2, 10)	6 (3, 12)	0.60
Nature of pain			0.14
Sore and Distended pain, n (%)	16 (59.3)	20 (74.1)	
Radiation pain, n (%)	2 (7.4)	4 (14.8)	
Needling pain, n (%)	2 (7.4)	0 (0)	
Other, n (%)	7 (25.9)	3 (11.1)	
Pain mode in 24 h			0.98
Gradually aggravated, n (%)	10 (37)	9 (33.3)	
Gradually relieved, n (%)	7 (25.9)	7 (25.9)	
No change, n (%)	6 (22.2)	6 (22.2)	
Other, n (%)	4 (14.8)	5 (18.5)	

Non-parametric test analysis was used for continuous variables and χ^2^ test was used for non-continuous variables. The numeric variables in the table were shown as median and Interquartile range.

Abbreviations: BMI, body mass index (calculated as weight in kilograms divided by height in meters squared); y, year; n, number; h, hour; 1 US dollar = 6.3734 ¥.

### Measures

Smoking status was evaluated by self-report by asking “Do you currently smoke every day, occasionally, or never?” Former smokers were excluded from the study. The rest of the participants were divided into “non-smokers” and “daily smokers.” The investigation neither differentiated former smokers from those who never smoked, nor specified the minimum quitting time to be considered as a non-smoker. The daily amount of cigarettes smoked by smokers was recorded. Health status under CLBP was assessed on a self-reported basis. Pain intensity was measured based on the Visual Analogue Scale (VAS) (18). The most severe pain intensity VAS_max_ and the mildest pain intensity VAS_min_ experienced by patients with CLBP were recorded. VAS is a commonly used pain scoring standard, and the pain intensity is divided into 10 points. 0 points indicate no pain, and 10 points indicate severe pain.

Anxiety and depression levels were measured based on the Self-Rating Anxiety Scale (SAS) and Zung Self-rating Depression Scale (SDS) (19). The higher their scores, the more severe the symptoms. The degree of physical dysfunction was measured by Oswestry Disability Index (ODI) and Roland‐Morris Disability Questionnaire (RMDQ) (20, 21). The LBP disability assessment scale is an important tool in the evaluation and rehabilitation treatment system of LBP. The commonly used LBP dysfunction assessment scales in the world are ODI and RMDQ. The higher the scores, the more severe the dysfunction. The degree of fear was measured using the Fear-Avoidance Beliefs Questionnaire (FABQ), including FABQ-work (FABQ-W) and FABQ-physical activity (FABQ-PA) (22). The higher the scores, the higher the degree of fear-avoidance beliefs. The quality of life was measured using the 36-Item Short Form Survey (SF-36). The unipedal stance test with eyes closed was also used to measure balance ability.

### Statistical Analyses

All data were analysed using IBM SPSS Statistics Software (version 26.0). Demographic data from smoking and non-smoking groups were compared using the χ^2^ test and the Mann–Whitney U-test, expressed as frequencies and medians. Non-parametric tests were used to compare non-normal distributions and experiments with small samples, and *p*-values less than 0.05 were considered statistically significant. The differences in VAS, ODI, RMDQ, FABQ, SAS, SDS, and SF-36 between the smoking and non-smoking groups were examined by Mann–Whitney U-test. The correlation between the amount of cigarettes smoked daily and VAS_max_, VAS_min_, ODI, RMDQ, the frequency of LBP per month, FABQ_total_, FABQ-W, FABQ-PA, SF-36, SAS and SDS was explored by linear correlation analysis.

Linearly correlated variables, such as the amount of cigarettes smoked daily, were further analysed by multiple linear stepwise regressions. In addition, age, income, education level, history of LBP, frequency of LBP per month, work time, leisure time, duration of first pain and duration of LBP per day were included as confounding factors. Multiple linear regression was used to control for confounding factors and examine the association between the amount of cigarettes smoked daily and pain intensity, disability, mood and quality of life. Ordinal logistic regression analysis was used to explore the correlation between the amount of cigarettes smoked and the impact of LBP on work and life (none, mild, moderate and severe).

## Result

### Smoking vs. Non-Smoking

**FIGURE 1:**
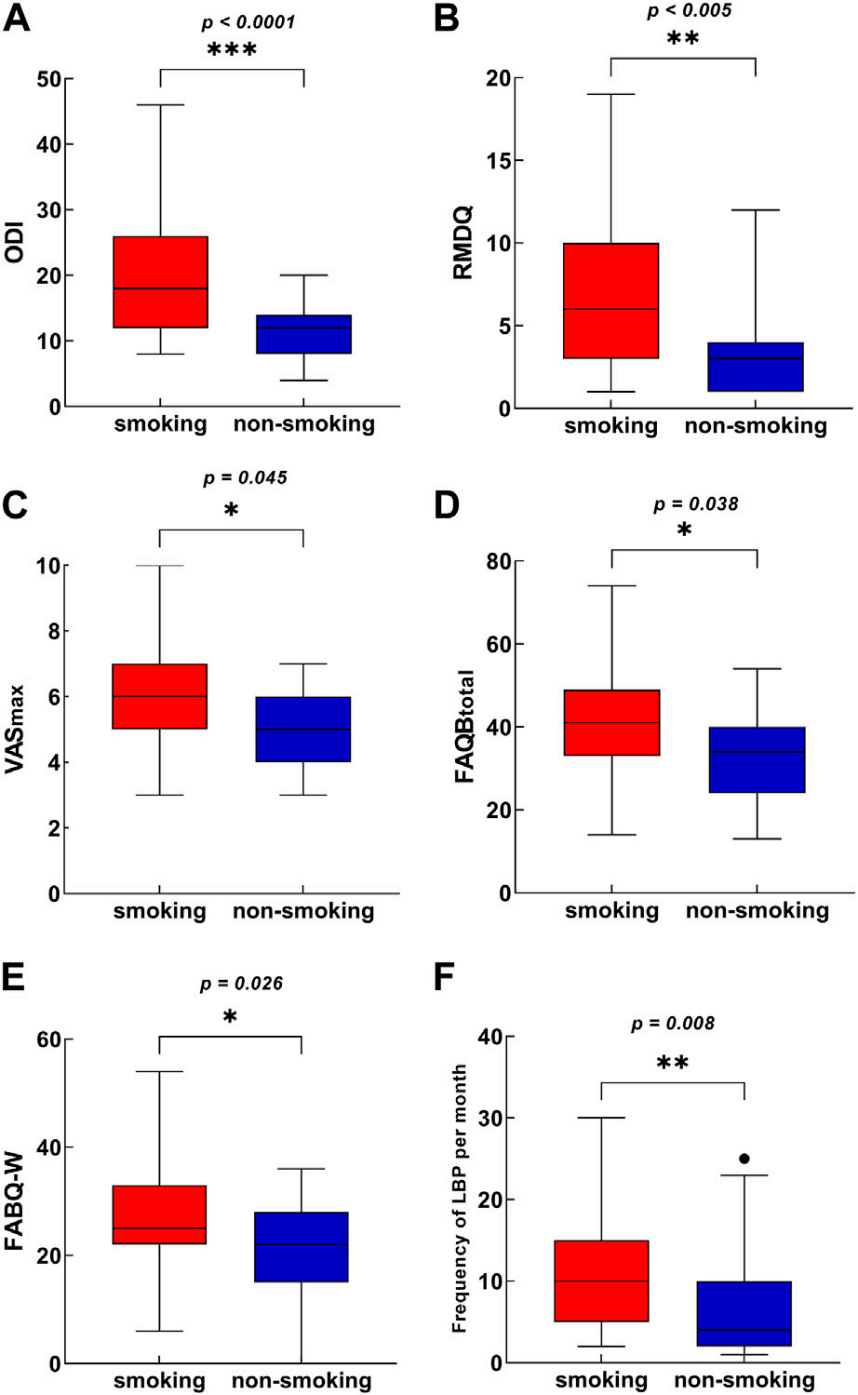
Difference in pain intensity and frequency, disability, and negative emotion outcomes between the smoking group and non-smoking group (Shanghai, China. 2018–2019). The Box-Whisker plot for each variable included the interquartile range and maximum/minimum values. **(A)** Difference in ODI between the CLBP smoking group and non-smoking group. **(B)** Difference in RMDQ between the CLBP smoking group and non-smoking group. **(C)** Difference in VAS_max_ between the CLBP smoking group and non-smoking group. **(D)** Difference in FABQ_total_ between the CLBP smoking group and non-smoking group. **(E)** Difference in FABQ-W between the CLBP smoking group and non-smoking group. **(F)** Difference in the frequency of LBP last month between the CLBP smoking group and non-smoking group. Abbreviations: ODI, Oswestry disability index; RMDQ, Roland‐Morris disability questionnaire; VAS, visual analogue scale; FABQ, fear-avoidance beliefs questionnaire; FABQ-W, FABQ work.

**FIGURE 2:**
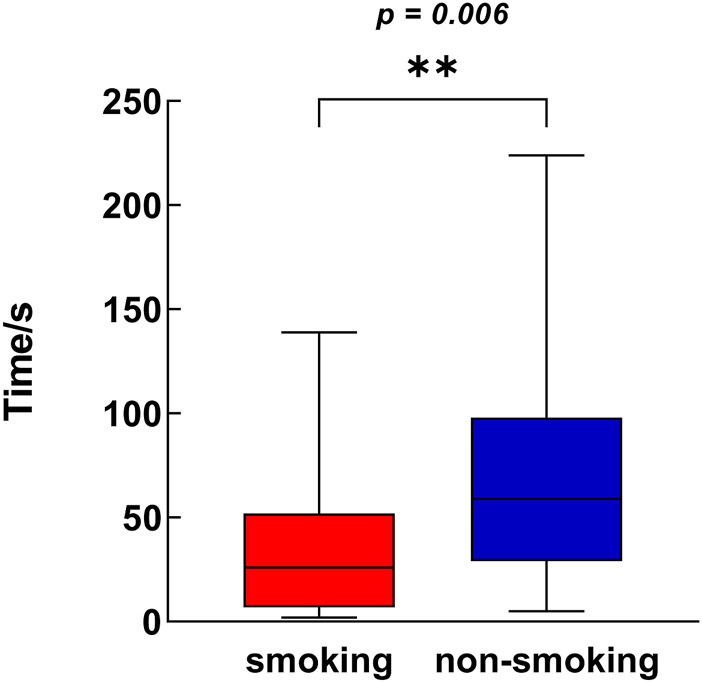
Difference in unipedal stance test with eye closed between smoking group and non-smoking group (Shanghai, China. 2018–2019). The Box-Whisker plot for each variable included the interquartile range and maximum/minimum values.

As shown in Figure 1, patients in the CLBP smoking group had worse average values on several measures than the non-smokers, which included VAS_max_, RMDQ, ODI, FABQ-W, FABQ_total_ and frequency of LBP per month of patients. However, no significant difference in VAS_min_, SAS and SF-36 was found between the smoking and non-smoking groups. The lack of difference in FABQ-PA between the smoking and non-smoking groups was consistent with the finding that no significant difference in the duration of moderate physical activity was observed between the two groups. The difference in FABQ between the two groups was primarily manifested in the fear-avoidance about work. The two groups also showed significant differences in the performance of the unipedal stance test with eyes closed, with smokers standing on one leg for less time than the non-smoking group (95% CI, 6.00 to 49.00; *p* = 0.0061; Figure 2). The impact of LBP on life (*p* = 0.006) and work (*p* = 0.032) was mostly moderate and severe in the smoking group, whereas it was mostly no effect or mild in the non-smoking group (Table 2).

**TABLE 2:** The impact of LBP on work and life in smoking group and non-smoking group (Shanghai, China. 2018–2019).

	Smoking group	Non-smoking group	
Work			*p* = 0.032
None, n (%)	3 (11.1)	2 (7.4)	
Mild, n (%)	10 (37)	20 (74.1)	
Moderate, n (%)	11 (40.7)	5 (18.5)	
Severe, n (%)	3 (11.1)	0 (0)	
Life			*p* = 0.006
None, n (%)	3 (11.1)	0 (0)	
Mild, n (%)	10 (37)	22 (81.5)	
Moderate, n (%)	12 (44.4)	5 (18.5)	
Severe, n (%)	2 (7.4)	0 (0)	

χ^2^ test was used for non-continuous variables. Abbreviations: n, number.

### Association Between the Daily Cigarette Smoking Amount and CLBP

**FIGURE 3:**
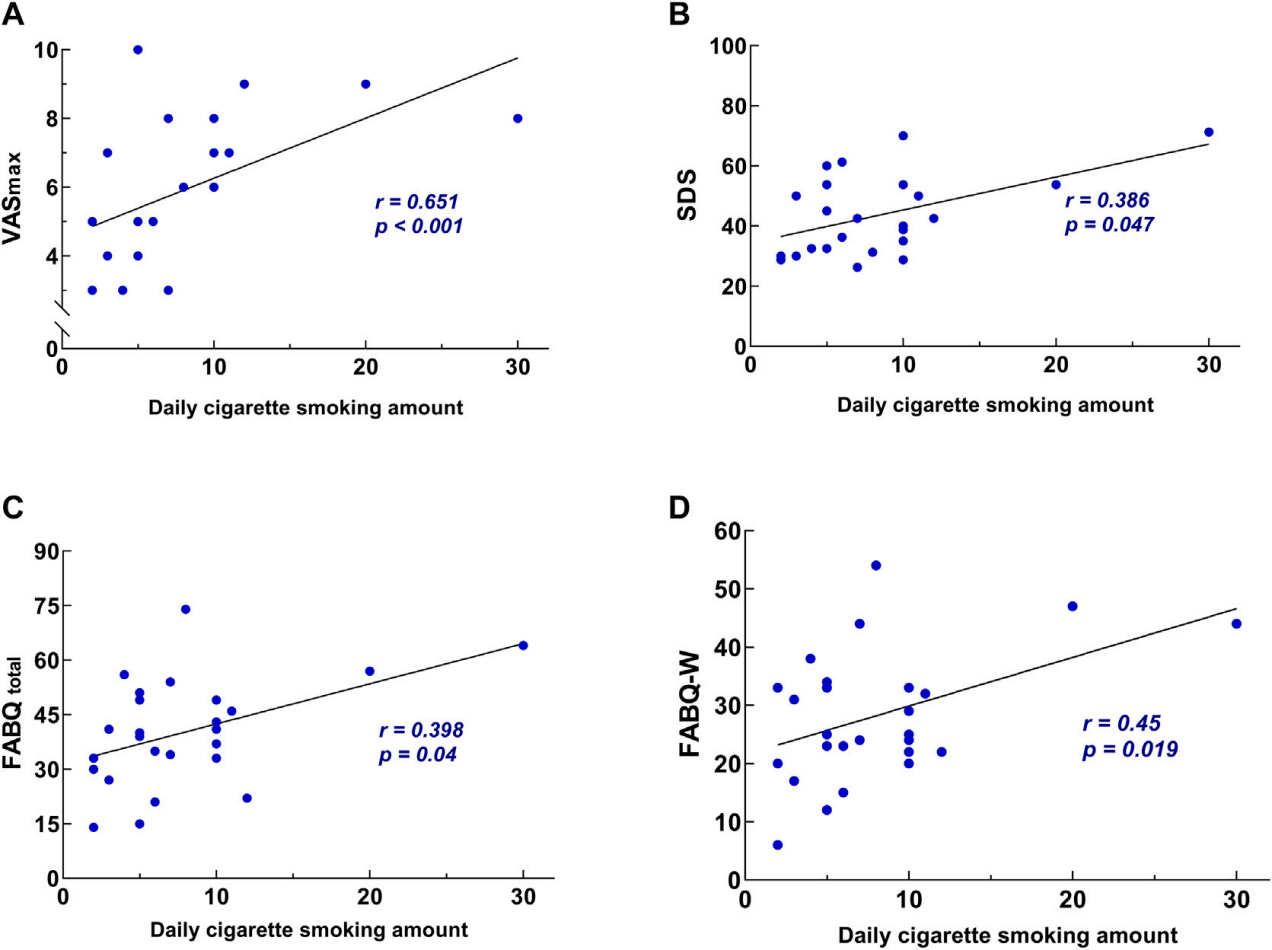
The correlation between the daily cigarette smoking amount and pain intensity, negative emotion outcomes (Shanghai, China. 2018–2019). **(A)** The correlation between the daily cigarette smoking amount and VAS_max_. **(B)** The correlation between the daily cigarette smoking amount and SDS. **(C)** The correlation between the daily cigarette smoking amount and FABQ_total_. **(D)** The correlation between the daily cigarette smoking amount and FABQ-W. Abbreviations: SDS, Zung Self-rating depression scale; VAS, visual analogue scale; FABQ, fear-avoidance beliefs questionnaire; FABQ-W, FABQ work.

A correlation was observed between the daily cigarette smoking amount and VAS_max_ (r = 0.651, *p* < 0.001), FABQtotal (*r* = 0.398, *p* < 0.040), SDS (*r* = 0.386, *p* = 0.047) and FABQ-W (*r* = 0.45, *p* = 0.019), but no relationship was observed between the amount of cigarettes smoked daily and ODI, RMDQ, VAS_min_, SF-36, FABQ-PA, the frequency of LBP per month and SAS (Figure 3). Multiple linear stepwise regression analysis indicated that the amount of cigarettes smoked daily had a statistically significant effect on VAS_max_ (b = 0.18, t = 3.25, *p* = 0.003), SDS (b = 1.10, t = 2.87, *p* = 0.008), FABQ-W (b = 0.84, t = 2.25, *p* = 0.02) and FABQ_total_ (b = 1.10, t = 2.60, *p*= 0.016). Considering that the partial regression coefficient values in multiple linear regression for different dependent variables are all positive, the increase in the amount of cigarettes smoked daily will lead to different degrees of increase in VAS_max_, FABQ-W, FABQ_total_ and SDS scores. The results of multivariate regression analysis indicated that after excluding the effect of other confounding factors, the amount of cigarettes smoked daily could independently be associated with pain, depressive symptoms and fear-avoidance belief in patients with CLBP. This result indicated that the increase in the daily cigarette smoking amount was positively correlated with the aggravation of pain intensity, depression and fear-avoidance belief in patients with CLBP.

The impact of LBP on work is an unavoidable *status quo* for patients with CLBP. Thus, the degree of work affected by CLBP is an important indicator of concern in this study. The effects of the amount of cigarettes smoked daily, moderate-intensity physical activity per week, age, leisure time, work time and economic income on this indicator were analysed using ordinal logistic regression with proportional odds assumption. Logistic regression analysis proved that the daily cigarette smoking amount was a significant predictor of the increasing impact of LBP on work (*p* = 0.019), which indicates its significant positive relationship with the impact level of LBP on work, that is, the higher the amount of cigarettes smoked daily, the greater the impact of LBP on the daily work. The OR value is 1.35 (95% CI, 1.05–1.73), which indicates that the amount of cigarettes smoked daily is an important factor leading to the aggravation of the impact of LBP on work. Amongst the factors related to the impact of LBP on life, the daily cigarette smoking amount did not show significance.

## Discussion

This study examined the association between smoking and pain, dysfunction, depression, anxiety and quality of life in patients with CLBP. We found significant differences in VAS, ODI, RMDQ and FABQ between smokers and non-smokers, which indicated that the smoking group had higher pain intensity, degree of dysfunction and fear-avoidance caused by CLBP than the non-smoking group. In addition, a correlation was observed between the daily cigarette smoking amount and pain, depression and fear belief in patients with CLBP.

The differences in pain and functional impairment between the smoking and non-smoking groups were statistically and clinically significant. VAS_max_ of the smoking group was concentrated at 5–7, whereas the non-smoking group was concentrated at 4–6. Therefore, patients with CLBP in the smoking group had severe pain, whereas the other group had mostly moderate pain (23). The clinical significance of the difference in ODI and RMDQ is significant. Patients in the smoking group likely have moderate disabilities, and they experienced more pain and difficulty sitting, lifting and standing (24). Patients in the non-smoking group had a mostly minimal disability, and they can cope with most living activities. However, no correlation was found between the daily cigarette smoking amount and ODI and RMDQ, indicating that the impact of smoking on the function of patients with CLBP can occur under slight smoking intensity and cannot necessarily aggravate with the increase of the amount of cigarettes smoked. The extent of the damage may be associated with the initiation and duration of smoking or the daily cigarette smoking amount at a particular stage in the evolution of the disease.

Regarding the association between smoking and pain, the results are consistent with previous studies (25). Smoking has been confirmed as a potential cause of musculoskeletal pain, and it is closely related to back pain (16). However, the mechanisms of LBP are only partly known. Smoking can increase the frequency of coughing, and coughing increases abdominal pressure, which intensifies the compression and stretch of the intervertebral disc on the nerve root, thereby blocking the venous return of the inflamed nerve root and increasing edema and sensitivity of the nerve to pain (26). Smoking is also associated with osteoporosis, which may alter the microscopic structure of the spine by reducing bone mineral content (27). It can impair fibrinolysis and increase fibrous deposition and scarring, leading to chronic infection and LBP (28). Moreover, smoking can reduce vertebral blood flow and affect intervertebral metabolic balance, thereby accelerating the degenerative process and making the spine more vulnerable to mechanical deformation and trauma (29–31). And it also reduces arterial blood flow, leading to ischemia of compressed nerve roots and pain. Furthermore, smoking alters disc gene expression, reduces collagen genes and increases proteoglycan and metalloproteinase 1 tissue inhibitory activity (32).

The amount of cigarettes smoked daily was correlated with VAS_max_ but not with VAS_min_. This result may indicate that the extent to which smoking affects chronic pain is related to its basic pain intensity. For slight pain conditions, the role of smoking as an influencing factor was not prominent. However, as the pain got worse, the association between smoking and pain also increased. Thus, this study may provide new insights into the mechanism of the association between smoking and pain. Later studies investigating the relationship and mechanism of smoking and pain can focus on further quantifying pain intensity and determining whether a non-linear correlation exists between smoking and pain intensity, that is, the greater the intensity of pain, the greater the association between cigarette smoking and pain intensity.

The performance of the two groups on the one-leg standing test was consistent with their pain intensity differences. CLBP reduces the stability of the spinal area and increases the activation time of the gluteus medius muscle, which leads to differences in the ability to control lower limb balance (33). Smoking may affect the ability of patients with CLBP to stand on a single leg by affecting their pain intensity. However, at present, no relevant research has been conducted to determine whether or not smoking directly affects the neural mechanism controlling muscle coordination, which may be the direction of future research.

This study also found that after adjusting for age, income, education level, history of LBP, frequency of LBP last month and other confounders, the daily cigarette smoking amount remained positively correlated with the SDS, which indicated the relationship between the amount of cigarettes smoked daily and the aggravation of depressive symptoms in patients with CLBP. The current study found that depression was correlated with smoking (34). Chronic pain reduces social connection, and smokers are less socially active and lonelier than non-smokers (35). Vogt et al. (36) adjusted for income, education level, occupation, employment, life stress, childhood adversity, divorce and neurosis and found that smoking remained associated with depression. Research suggests that the smoking–depression association is bipolar. The presence of depressive symptoms increases the risk of developing nicotine dependence in smokers, thereby increasing the risk of depression (37). The occurrence of smoking addiction and depressive symptoms has a common neurotransmitter pathway, and they may have a common genetic material basis (38).

Anxiety and depression have been associated with a bidirectional effect of promoting smoking (39), and negative emotions have been confirmed as a central mechanism for the correlation of pain with smoking (40, 41). Pain-related anxiety was relevant to increasing pain intensity and positively related to heavy smoking and nicotine use to cope with aversive states (42). Increasing pain-related anxiety and pain sensitivity were related to early initiation of smoking (43). Whether or not smoking had a significant effect on the anxiety of patients with CLBP was not shown in this study probably because anxiety caused by the pain intensity of CLBP was not enough to cause the related effect of smoking, or the negative emotions caused by CLBP were more prone to depression. Future treatments should target whether CLBP-related anxiety may contribute to behavioural tendencies to relieve pain and negative effects through cigarette smoking.

CLBP can be attributed to a biological–psychological–social phenomenon. Patient’s anatomical injury factors interact with psychosocial factors (44), such as fear-avoidance belief, which indicates that some patients with LBP have negative beliefs about pain that can lead to a catastrophic psychological reaction, causing the patient to fear activities that may aggravate pain or injury. Avoidance of such activities can reduce the likelihood of repeat pain or injury (45). Fear-avoidance beliefs can be a predictor of outcome in patients with subacute LBP (46). In this study, the daily cigarette smoking amount was found to be positively correlated with FABQ_total_ and FABQ-W, particularly to work, which in turn led to excessive fear of pain or injury and gradually extended to fear of physical movement. This finding is also consistent with the association between smoking and pain intensity and depression in this study. Depression is an important influencing factor of fear-avoidance beliefs (47). Considering that depressed patients are often in a state of low emotional responsiveness and lack of enthusiasm and motivation to actively respond to symptoms, they tend to develop fear-avoidance beliefs about pain. Depression, including clinically diagnosed depression and patient-reported depressive symptoms, is common in patients with CLBP (48). Pain patients with depressive symptoms tend to have more intense pain experiences and more severe physical damage. Smoking is closely related to depression (37), indicating that smoking may be related to fear beliefs by affecting depressive symptoms in patients with CLBP. Future research can focus on the interaction of smoking, depression and fear-avoidance beliefs in patients with CLBP.

The daily cigarette smoking amount is related to the impact of LBP on work but not related to the impact of LBP on life probably because the working conditions of most people are relatively monotonous, which reduces the impact of other factors to a higher degree, thereby making the impact of smoking fully exposed. In daily life, individuals have more options to maintain low-intensity LBP for themselves, which highlight the weight of factors such as income, leisure time and daily activity in the impact of LBP on life and indirectly offset the effect of the amount of cigarettes smoked daily. This result indicates that future research should include more population in the study to explore the correlation between smoking and the impact of LBP on life under more undisturbed conditions.

### Conclusion

This cross-sectional study investigated the association between smoking and pain, dysfunction and psychological status in patients with CLBP and analysed the relationship between the amount of cigarettes smoked daily and CLBP. The results of the study indicated that smoking was related to the aggravation of symptoms in patients with CLBP, which suggested that patients with CLBP and people at risk of LBP should be aware of the harm caused by smoking. Given the limitation of the sample size and cross-sectional study, this study cannot explain the corresponding causation between the amount of cigarettes smoked daily and the aggravation of CLBP. Future research should further expand the sample size and control economic conditions, medical level, occupation and other confounding factors.
